# Characterization and effectiveness of pay-for-performance in ophthalmology: a systematic review

**DOI:** 10.1186/s12913-017-2333-x

**Published:** 2017-06-05

**Authors:** Tim Herbst, Martin Emmert

**Affiliations:** 1 nordBLICK Augenklinik Bellevue, Lindenallee 21-23, 24105 Kiel, Germany; 2 Friedrich-Alexander-University Erlangen-Nuremberg, School of Business and Economics, Institute of Management (IFM), Lange Gasse 20, 90403 Nuremberg, Germany

**Keywords:** Pay for performance, P4P, Ophthalmology, Systematic comparison

## Abstract

**Background:**

To identify, characterize and compare existing pay-for-performance approaches and their impact on the quality of care and efficiency in ophthalmology.

**Methods:**

A systematic evidence-based review was conducted. English, French and German written literature published between 2000 and 2015 were searched in the following databases: Medline (via PubMed), NCBI web site, Scopus, Web of Knowledge, Econlit and the Cochrane Library. Empirical as well as descriptive articles were included. Controlled clinical trials, meta-analyses, randomized controlled studies as well as observational studies were included as empirical articles. Systematic characterization of identified pay-for-performance approaches (P4P approaches) was conducted according to the “Model for Implementing and Monitoring Incentives for Quality” (MIMIQ). Methodological quality of empirical articles was assessed according to the Critical Appraisal Skills Programme (CASP) checklists.

**Results:**

Overall, 13 relevant articles were included. Eleven articles were descriptive and two articles included empirical analyses. Based on these articles, four different pay-for-performance approaches implemented in the United States were identified. With regard to quality and incentive elements, systematic comparison showed numerous differences between P4P approaches. Empirical studies showed isolated cost or quality effects, while a simultaneous examination of these effects was missing.

**Conclusion:**

Research results show that experiences with pay-for-performance approaches in ophthalmology are limited. Identified approaches differ with regard to quality and incentive elements restricting comparability. Two empirical studies are insufficient to draw strong conclusions about the effectiveness and efficiency of these approaches.

**Electronic supplementary material:**

The online version of this article (doi:10.1186/s12913-017-2333-x) contains supplementary material, which is available to authorized users.

## Background

Remuneration systems applied within national healthcare systems are frequently discussed and criticized [1–3]. A central point of discussion is that physicians and medical institutions are paid for their services without incorporating the provided quality of care. During recent years, the implementation of pay-for-performance programs (P4P programs) has become a popular tool to foster quality improvements in healthcare [4, 5]. Public and private initiated pay-for-performance programs pursue this goal by linking financial payments to the achievement of predefined quality targets [6].

Various systematic reviews have summarized existing P4P programs and evaluated their empirical evidence based on different criteria [7–15]. With regard to changes in the quality of care after implementing P4P, improvement effects were primarily apparent. Although a number of studies showed clear quality improvements [16–21], a few studies found no quality effects [22, 23] or even negative quality effects [24]. Similar unambiguous results were summarized for cost effects. While some studies resulted in positive cost effects (cost savings, cost efficiency) [18, 21, 24–27], others found increased costs after P4P implementation [16, 20, 22]. The mixed results appear to have multiple causes, such as differences in the P4P program structure and the design of the empirical studies, which limit the comparability of the results [9–11, 13]. Nevertheless, several studies found the choice of quality measures and the design of the incentive structure to be crucial for the success of P4P programs [9, 11, 28, 29].

Little is known regarding P4P programs in ophthalmology and the available empirical evidence. To elucidate the existing P4P approaches for this medical specialty, a systematic review was conducted, which pursued the following objectives: 1) to provide an comparative overview of existing P4P programs in ophthalmology; 2) to compare the identified P4P approaches by applying the *Model for lmplementing and Monitoring lncentives for Quality* (MIMIQ) by Van Herck et al.; and 3) to summarize their empirical evidence, particularly with regard to the quality and cost effects.

## Methods

In this review, a systematic search of existing literature published between January 2000 and May 2015 was conducted. Studies written in English, French, or German have been included, and the review complied with the Guidelines from the Cochrane Collaboration [30].

First, the following databases were searched: PubMed, the NCBI website, Scopus, the Web of Knowledge, EconLit, and the Cochrane Library. In addition, reference lists of identified systematic reviews were screened for additional empirical papers.

As a second step, a systematic internet search was conducted to identify additional P4P programs. Google and Google Scholar were used to identify additional papers, studies, articles, and initiatives concerned with P4P in ophthalmology. The websites of several governmental institutes and healthcare insurances (e.g., the *Institut für Qualität und Wirtschaftlichkeit im Gesundheitswesen (*IQWIG) and the National Health Service, UK) were scanned for additional P4P initiatives and for further information regarding already identified P4P programs. Finally, experts were contacted to receive further indications of ongoing or finished studies.

The following search terms have been selected on the basis of *Mehrotra* et al. [14] and further extended: *ophthalmology* and *pay-for-performance, paying for quality, P4P, physician incentive, incentive payment, pay for value, pay for quality, payment for quality, value-based purchasing, performance-based payment, performance-based reimbursement, performance-based pay, output-based payments, output-based pay, incentive reimbursement, incentive program, quality-based purchasing, financial incentives, quality and bonus, quality and reward, or monetary incentive.*

The titles and abstracts of potentially relevant studies were judged against the predefined inclusion and exclusion criteria (see below). If a paper appeared to comply with the criteria, a full text version has been supplied to judge whether to include it in this review.

### Inclusion and exclusion criteria

In general, empirical studies as well as purely descriptive papers were included. We utilized controlled clinical trials and randomized controlled studies as well as observational studies. The studies were further required to measure at least one of Donabedians` quality dimension (the structure, process, or outcome quality) [31] as the basis for compensation. Both positive (bonus) and negative (penalty) types of financial incentives were accepted. Variable, as well as fixed amount, payments were approved. News reports, presentations, and recommendations as well as comments were excluded.

### Study Scoring

To assess the methodological quality of identified empirical studies, Critical Appraisal Skills Programme (CASP) checklists were used [32]. Depending on the study design, eight different checklists are available including 10 to 12 questions grouped into three checklist-depending sections. The score possibilities for each checklist criterion were 1 (checklist criterion satisfied) and 0 (checklist criterion not satisfied, or unclear) [33]. The total scoring (percentage value) was calculated for each empirical study by dividing the total amount of the achieved points by the maximum number of achievable points. The scoring calculation was conducted by the authors (TH, ME).

### Systematic characterization of the identified pay-for-performance approaches

Characterization of the identified P4P approaches was conducted according to the MIMIQ developed by van Herck et al. [34]. The model provides an overview regarding the crucial factors for successfully establishing a P4P program and defines 23 steps that should be considered when developing and implementing a P4P program. Characterization of P4P programs will be carried out by applying the 14 steps concerned with quality and incentives.

## Results

**Fig. 1:**
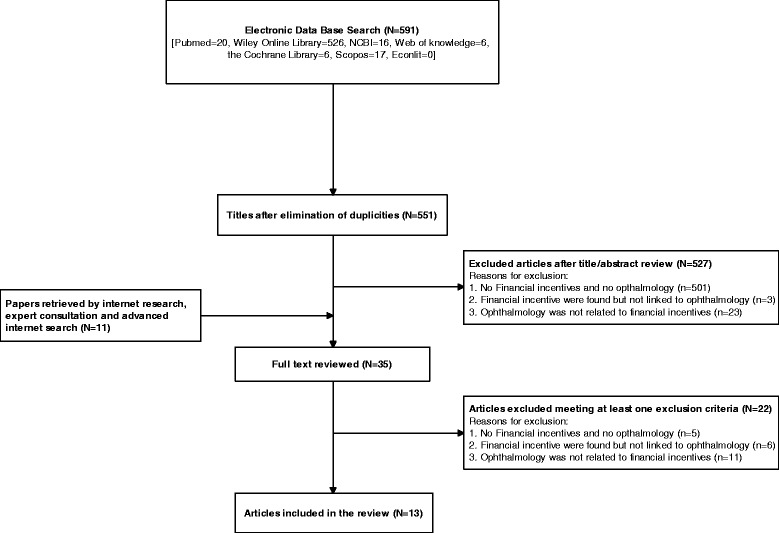
search flow and results A systematic review of published literature was conducted in electronic data bases. After elimination of duplicates, title and abstracts of the remaining papers were reviewed. 24 full texts were reviewed leading to 13 articles, which were finally included

According to the predefined search terms, 591 potentially relevant papers were identified in the electronic databases. After eliminating duplicates and judging the titles and abstracts, 24 papers were retained for assessment of the full-text version. An overview of the detailed reasons for exclusion is presented in Fig. 1. Advanced internet research, expert consultation, and an advanced internet search provided 11 additional papers. After reading and assessing the remaining 35 articles, two empirical studies and 11 descriptive papers met the predefined inclusion criteria. In total, 13 identified papers led to the identification of four pay-for-performance programs concerned with ophthalmology (see Fig. 1 and Additional file 1).

The *MedEncentive Information Therapy Program* is a web-based incentive system that rewards the primary care physician as well as the patient for adhering to evidence-based medicine (EBM) guidelines and a healthy lifestyle. This P4P approach, first introduced as a pilot program in Duncan, Oklahoma in August 2004, pursues the aim of enhancing communication between the physician and patient via a web-based platform named MedEncentive [26] (Table 1).

**Table 1:** Key characteristics of identified pay-for.performance programs in ophthalmology

	Year of Implementation	Countries	Quality measurements	Object of financial incentives	Empirical studies	Study Score^a^
MedEncentive	2004	USA (City of Duncan)	Reporting Quality Compliance with guideline-based treatment recommendations Compliance with physicians instructions Patient satisfaction	Physician Patient	1 study: introduction of P4P led to less global expenditures	45.8%
Kaiser Permanente Northern California	1999	USA (California)	Screening rate for diabetic retinopathy	Medical facility	1 study: Removal of financial led to a decrease in screening rate for diabetic retinopathy	71.4%
Physician Quality Reporting System (PQRS)	2006	USA	Reporting quality	Physician	n.a.	n.a.
ProvenCare	2006	USA	Compliance with best practise guidelines for cataract surgery	n.a.	n.a.	n.a.

^a^Methodological quality of a study (study score) was measured according to Critical Appraisal Skills Programme (CASP) checklists [32]

Regarding the *quality aspects*, MedEncentive focuses on the cost effectiveness of the quality of health services. The platform aimed for high patient satisfaction and centeredness. Licensed health plans and self-insured employers (per member per month fee) were able to participate. Twenty ophthalmic outcome indicators covered the process and outcome quality. Based on the ICD-9 diagnosis code, MedEncentive searches for appropriate EBM guidelines, which were proposed to the treating physician via email. The measurability of SMART criteria (specific, measurable, achievable, relevant, and timely) was restricted because of the nominal scale level of the quality indicators. The risk adjustment was taken into consideration in the cost calculation (e.g. for “catastrophic cases”) [26] (Table 2).

**Table 2:** systematic comparison of *quality elements* according to van Herck et al

	Quality of health services	Patient population	Structure, process and outcome indicators	Underuse and/or overuse	Number of targets and indicators	Best practise and SMART criteria^a^	Risk adjustment of outcome measurement
MedEncentive	- cost effectiveness - Patient satisfaction and centeredness	- licensed health plans - self-insured employers	- process quality - outcome quality	- based on EBM guidelines - targets overuse reduction	20	- SART - nominal scaled quality indicators (no metric QI)	Risk-adjustment for cost calculation
Kaiser Permanente Northern California	- patient safety	- Age > 30 years - restricted to diabetic retinopathy screening	- process quality	- Risk of overuse (screening for diabetic retinopathy)	1	- SART - nominal scaled quality indicators (no metric QI)	- age-related risk adjustment
Physician Quality Reporting System (PQRS)	- Clinical quality measurements - Equity and timeliness	- Medicare & Medicaid	- process quality - outcome quality	-EBM guidelines	11	- SMART	- not clear
ProvenCare	- cost effectiveness - Equity and timeliness	- restricted to cataract surgery	- process quality - outcome quality	n.a.	40	- SMRT (no information concerning achievability)	- n.a.

^a^SMART means specific, measurable, achievable, relevant and timely

Concerning the *incentive elements*, the bonus and penalty payments were defined as the “absolute rewards”. The physicians were financially rewarded for transcribing information via MedEncentive regarding the applied patient-based therapy, which might deviate from recommended EBM guidelines. When physicians approved treatment according to EBM guidelines, or a reason for deviation was mentioned, the reimbursement increased by 10%. Reimbursement decreased if physicians rejected the proposed treatment without valid justification [26, 35]. The patient’s remuneration was determined primarily by compliance with physician’s orders. By reading diagnosis-related information, therapy information, and answering questions regarding his/her personal health status and level of satisfaction with the physician’s performance, the patients could be financially rewarded, which was reflected in reduced co-payments [35]. No information was available regarding the frequency of payments (Table 3).

**Table 3:** systematic comparison of *incentive elements* according to van Herck et al

	Incentive structure	Incentive size	Relation between Incentive structure and quality achievements	Frequency of incentive payment	Duration of incentive payments	Relative weights for quality indicators	Form of incentive structure
MedEncentive	Bonus Penalty	10% (bonus) n.a. (penalty)	Absolute Reward	n.a.	Since 2004	n.a.	Fixed amounts
Kaiser Permanente Northern California	Bonus	n.a.	Absolute Reward	n.a.	Since 1999	No relative weights	Fixed amounts
Physician Quality Reporting System (PQRS)	Bonus Penalty	+0.5%-to +2.0% (bonus) −1.5% (penalty)	Absolute Reward	n.a.	Since 2006	Yearly adjustment of indicators and amount of payments	(Fixed amounts)
ProvenCare	Bundled payments	n.a.	Absolute Reward	n.a.	Since 2006	n.a.	Fixed amounts

In 1999, Kaiser Permanente of Northern California introduced a pay-for-performance system in 35 of their medical facilities. For 20 clinical quality indicators, the regional operations leadership specified financial incentives for reaching predefined target goals. Financial incentives were paid to medical facilities, which could autonomously determine the use of the funds (funding core facilities, staffing, and quality improvements). Concerning ophthalmology, screening for diabetic retinopathy was included as one quality indicator [36] (Table 1).

With regard to *quality elements,* patient safety was defined as one quality aim. According to recommendations of the American Diabetes Association and the American Academy of Ophthalmology, screening for diabetic retinopathy should begin regularly for patients at the age of 31 years [37, 38]. The quality requirements for diabetic retinopathy screening were considered to be met if a patient’s visit to the optometry or ophthalmology department was recorded within 2 years after the end of the assessment year. In best practice and SMART criteria, measurability was fulfilled to a limited extent because of the nominally scaled quality indicator. Risk adjustment of the outcome measurement was partially implemented because the target group was limited to a minimum age of 31 years (Table 2).

Bonus payments (*incentive elements)* were implemented in 1999 as absolute top payments, which linked a possible bonus payment to the undertaken screening for diabetic retinopathy. Since only one quality indicator was used, no relative weights were defined. The bonus payments were defined as fixed amounts (Table 2).

The *Physician Quality Reporting System* (PQRS*)* 1 is an American P4P approach, established by the Centers for Medicare & Medicaid Services (CMS) in 2006. During the first year, the lack of financial incentives for data collecting and reporting resulted in low participation. With the Tax Relief and Health Care Act passed by Congress in 2006, financial rewards for participating physicians were enabled, which increased the participation in PQRS [26]. In 2011, more than 280,229 physicians representing various medical disciplines have participated, receiving more than $261,000,000 (US) [39] (Table 1).

Regarding the *quality elements*, PQRS focused on clinical quality as well as on equity and timeliness. The quality measures were developed by discipline-specific societies, analyzed by the American Medical Association Physician Consortium for Performance Improvement (AMA-PCPI) and endorsed by the National Quality Forum (NQF) [40]. The inclusion and exclusion of quality measures was conducted at regular intervals. In 2013, 203 quality measures^2^ for claims were defined, of which 11 measures (e.g., cataracts, age-related macular degeneration and diabetic retinopathy) were allocated to ophthalmology [41, 42]. Detailed information regarding the risk adjustment of outcome measures was not presented (Table 2).

*Financial incentives* were offered by bonus and penalty payments. To receive bonus payments, physicians must report three different measures for at least 80% of their fee-for-service Medicare patients during the settlement period. The incentives were paid as “absolute rewards” [43]. In 2006, the level of bonus payments for successful data reporting started with 1.5% of the physician’s Medicare billings and increased to 2.0% in 2008. In 2011, a gradual decrease in incentive payments to 1.0% was continued with another decrease to 0.5%. The introduction of a penalty payment of 1.5% in case reporting criteria is not met is planned for 2015 [44] (Table 3).

*ProvenCare®* is an alternative P4P approach for selected operations, which was founded by Geisinger Health Care (Danville, Pennsylvania). The “90-day warranty,” a key feature, guarantees cost coverage for the surgery as well as for follow-up treatments within 90 days [45]. The program started in 2006 with elective bypass surgery (a coronary artery bypass graft, called ProvenCare® CABG) and expanded its portfolio with total hip replacement, percutaneous coronary intervention (PCI), perinatal treatment, and bariatric surgery [46]. In June 2006, Geisinger Health Care began testing its P4P approach for cataract surgery [47] (Table 1).

Regarding the definition of the quality of health service according to Campbell et al. [48], the cost effectiveness as well as equity and timeliness were followed by the P4P approach. The target group was restricted to cataract patients [46]. Program implementation occurred in six stages. In the first phase (*Engage Champion*), a discussion of the entire program occurs in detail. The second phase (*Compile Evidence*) includes literature review and guidelines, which leads to the definition of best practices (*Establish Best Practices*). In the following phase, *Establish Components/Redesign*, the redesign of existing processes and workflows occurs before the beta testing phase begins, in which the data collection for the final modification occurs (*Go Live Beta*). The final phase of the process engagement represents the completion of the redesign, which leads to the *full deployment phase* [47]. Forty pre- and post-operative quality indicators measured the process and outcome quality. However, no detailed information regarding risk adjustments was available [45] (Table 2).

Concerning the incentive elements, ProvenCare used performance-based bundled payments, including hospital and physician payments, which was the striking incentive element of this approach. The program acts as a warranty because potential follow-up treatments within 90 days after surgery are covered [45, 49]. No detailed information concerning the size and frequency of the incentive payments was available (Table 3).

### The effect of pay-for-performance on the quality of care: the empirical evidence

With regard to the empirical evidence of the identified P4P approaches, papers on two different approaches were identified during the systematic search procedure*. Lester* et al. evaluated, by means of a longitudinal analysis, the effects of removing financial incentives on the performance level of four clinical quality indicators in 35 outpatient facilities of Kaiser Permanente Northern California. In addition to screening for diabetic retinopathy, the assessment of the patient level of glycemic control (HbA1c <8%), control of hypertension (systolic blood pressure < 140 mmHg), and screening for cervical cancer were enrolled. Besides gaining empirical insights into the effects of removing financial incentives on performance levels, the authors were motivated to conduct this study because of the political decision to remove eight quality indicators from the Quality and Outcome Framework in April 2011. The authors expected that removing the financial incentives would lead to a significant decrease in performance levels. Overall, 2,523,659 adult patients were included in the analyses. Following the removal of financial incentives 5 years after implementation, changes in the rates of screening for diabetic retinopathy were analyzed over a total period of 9 years (1999 to 2007). Hierarchical regression models were used to estimate the effect of removing the financial incentive on the annual change in the screening rate. For the five consecutive years with financial incentives, the screening rate rose from 84.9 to 88.1%. Following the incentive removal, the screening rate decreased to 80.5% after 4years. In year-to-year changes at the facility level, removing financial incentives led to an average decline of 3% per year. [36] Applying the Critical Appraisal Skills Programme (CASP) checklists, a score of 71.4% was calculated (Table 1).

In a second paper, *Parke* examined whether global health care expenditures might decrease by establishing MedEncentive. Empirical data were collected within the framework of a pilot study, established in the city of Duncan in August 2004. A retrospective cost analysis, which compares global expenditures of implementation relative to the baseline year, was conducted. The results show a global cost reduction of approximately 11.5%, from $2,316,929 (baseline year) to $2,049,780 (implementation year). With regard to the disease category of the nervous system and sensory organs (ICD-9: 320-389) and covering ophthalmologic diseases as well, the total expenditures decreased from $94,476 (baseline year) to $77,715 (implementation year). This finding corresponds to a cost reduction of $16,761 (17.7%) [26]. The study score was determined to be 45.8% (Table 1).

## Discussion

The aim of this study was to provide an overview of the existing pay-for-performance approaches in ophthalmology and their empirical evaluations. A general comparison according to key characteristics showed that the four identified P4P approaches were implemented in the USA between 1999 and 2004. With regard to the underlying quality measurements and objectives of the financial incentives, differences between the four approaches were identified.

A systematic characterization and comparison of the quality and incentive aspects was conducted according to van Herck et al. [34] With regard to the quality dimensions, three approaches measured the process and outcome quality, whereas the approach of Kaiser Permanente measured solely the process quality (Table 2). In their review, *Van Herck* et al. concluded that the improvement rates of the assessed studies were higher for the process measurements than for the outcome measurements [13]. With regard to efficiency, *Emmert* et al. did not draw this conclusion, although the implementation of the outcome measures into practice appear to be more difficult than implementing the process measurements [11]. *Campell* et al. [48] mentioned that the P4P approaches should focus simultaneously on several quality dimensions. With regard to the number of quality targets and indicators, the corresponding values of the identified approaches ranged between 1 (Kaiser Permanente) and 40 (ProvenCare). While too few indicators might lead to a disproportionate focus on rewarded behavior, too many indicators might result in an unintended complexity, whereby the stimulation of desired behaviors might fail to appear [50].

With regard to the incentive elements of the P4P approaches, the incentive size ranged from +10% to −1.5%. The financial incentives were implemented as bonus and penalty payments (MedEncentive, PQRS), bonus payments (Kaiser Permanente) and performance-based bundled payments (ProvenCare), which were predominantly paid as individual incentives. In his review, *Eijkenaar* found several papers that examined the pros and cons of individual versus group incentives. The performance measurements on the individual physician-level frequently failed because of small patient panels [51, 52] or less variation in quality measures compared to facility-level measurements, which might lead to inaccurate remuneration [53]. Group-level-based performance payments might force undesirable behavior of single group members, such as “free riding” [50, 54]. In their review, *Frølich* et al. compared, among others, group vs. individual recipients. Among the studies targeting individual recipients, the quality of five measurements increased, whereas two measurements showed a decreased quality. With group recipients, the quality of one measurement increased whereas two measurements showed opposite effects [55].

The main criticism of the identified P4P approaches was managing the incentive elements and their effect on the quality of care. In the context of PQRS, participating physicians were rewarded for data reporting and not for achievements of certain quality requirements. Therefore, the influence of financial incentives on the quality of medical care appeared to be questionable. *Federman and Keyhani* supported this suspicion in a survey of PQRS participants. Overall, 50.1% of the responding physicians were of the opinion that the PQRS program had no effect on the quality of care; 36.1% answered that it had a small effect, and 13.8% assessed the effect as moderate or large [56]. With regard to ProvenCare, the separation of payments for interdisciplinary treatments (e.g., diabetes) and the considerable administrative efforts were the main criticisms. The bundled payments could lead to a “cost-containment mechanism” if corresponding amounts are left unchecked at regular intervals [49].

The identified empirical studies showed that the implementation of a P4P program might lead to cost reductions [26]. *Parke* demonstrated in his financial analysis that MedEncentive might lead to cost reduction after 1 year compared to the baseline year. In the ophthalmology field, an isolated cost effect could not be determined because the relevant ICD-9 range could not be solely assigned to eye diseases. The author justified the omission of these analyses with a subsample that was too small to provided statistically significant results. With regard to the examined ICD-9 ranges, described cost effects include the volume effects, which might have occurred within a single ICD-9 disease (intra-specific) and/or between different ICD-9 diseases (inter-specific). The inter-specific changes in the number of ICD-9 ranges before and after the MedEncentive implementation were calculated. However, their influences on the described cost effects have only been justified with the omission of unnecessary treatments. With a self-calculated study score of 45.8%, the main weaknesses are attributable to the “economic evaluation” and “cost assessment and cost comparison” sections.

In the context of Kaiser Permanente Northern California, *Lester* et al. showed that the presence/absence of financial incentives might result in quality improvements /deteriorations [36]. Unfortunately, the levels of significance for the annual changes in the screening rate before and after the removal of financial incentives were not calculated. Further points of weaknesses mentioned by the author are the lack of a control group and the type of financial incentives used. Financial rewards were addressed to the medical facility and not directly to the physician. As a consequence, the effect of incentives on a physician’s behavior is questionable. Other possible factors (e.g., computerized reminders) that might have had an influence on the screening rate for diabetic retinopathy were not examined. A final conclusion on the effects of the financial incentives on the screening rate was possible only to a limited extent. Regarding the study scoring of 71.4%, the main points of criticism are related to the result section.

Neither of the two studies examined the effect of the P4P programs on cost and quality development simultaneously. With the help of different economic evaluations of P4P programs, *Emmert* et al. showed in their systematic review that quality improvements are frequently coupled with higher costs [11]. To draw valid conclusions regarding this aspect, a simultaneous examination of the costs and quality effect would have been desirable in both studies.

In order to achieve the goals of effectiveness and efficiency improvement, future P4P approaches should pay more attention to a well-established design of underlying quality measures and incentive structure. Although evidence about the optimal design of single P4P elements is mainly lacking or the subject of controversial discussion, several generally applicable recommendations can be made on the basis of the available literature. In order to promote awareness for the approach, providers should be involved in the process of program design. A broad set seems to be favorable for quality measures, which is aligned with professional norms and aims for quality attainments and quality improvements [9, 28, 29, 50, 57]. Financial incentives should at least compensate for additional costs related to measures taken for quality improvements. Furthermore, quality-based payments should be sufficiently large to motivate behavioral responses [9, 29, 58, 59]. Since individuals tend to value immediate earnings more than future earnings, small, frequent payments are preferable to a single, large lump sum payment [60].

The applicability of these general P4P requirements to the field of ophthalmology seems to be possible without major restrictions. The implementation of requirements on financial incentives and payment frequency depends primarily on the financial provider and less on ophthalmology as a medical specialty. Regarding the requirement of a broad set of quality measures, identified P4P approaches show that cataract surgery already offers at least 40 possible quality measures related to process and outcome quality [45]. Further quality measures seem to be definable regarding the whole field of ophthalmology. In addition to screening for diabetic retinopathy, further disease-specific diagnostic requirements may be specified in order to capture process quality. Ophthalmology offers several measurable findings (e. g. visual acuity (decimal), intraocular tension (mmHg), refraction (dioptre)), which may be used in order to assess outcome quality. Future P4P approaches should take these recommendations more into account when designing P4P incentive schemes which aim to improve both the effectiveness and efficiency in ophthalmology and other disciplines.

## Conclusions

In total, four P4P approaches have been identified in ophthalmology, of which two were evaluated within empirical analyses. The comparison of the framework setting showed numerous differences between the identified P4P approaches, which might have influenced the performance of the P4P approaches. Because of the lack of empirical analyses, clear conclusions regarding their relative advantages and disadvantages could not be drawn. Further empirical analyses of P4P approaches appear to be necessary to assess their influences on the quality and cost of healthcare in general and in ophthalmology in particular.

### Limitations

This review has several limitations. The inclusion criterion that P4P programs have to include (quality) measures that can be assigned to at least one of Donabedian’s quality dimensions may lead to the impression that aspects like cost effectiveness or patient satisfaction may have been neglected during the screening process. Performance is a multi-dimensional construct, which can be conceptualized in different ways. However, our literature screening results are robust since we did not exclude any study for using quality measures that were not captured under Donabedian’s quality triangle. Furthermore, the aim of this study was to identify various pay-for-performance approaches in ophthalmology and their empirical evaluation. Because this medical discipline might be part of interdisciplinary P4P approaches, identification of further relevant approaches might have been missed. A comparison of the identified empirical studies facilitated by the calculated study scores is challenging because the studies differ in the type of evaluation.

## Additional file

Additional file 1: First results of search history (including duplicates), separated for single keyword combinations and for single databases. (DOCX 15 kb)

## Abbreviations

AMA-PCPI: American Medical Association Physician Consortium for Performance Improvement (AMA-PCPI)
CABG: Coronary Artery Bypass Graft
CASP: Critical Appraisal Skills Programme
CMS: Centers for Medicare & Medicaid Services
dec.: Decimal
dpt.: Dioptre
ICD: International Classification of Diseases
IQWIG: Institut für Qualität und Wirtschaftlichkeit im Gesundheitswesen
MIMIQ: Model for Implementing and Monitoring Incentives for Quality
mmHg: millimeter of mercury
NQF: National Quality Forum
P4P: Pay-for-Performance
PCI: Percutaneous Coronary Intervention
PQRI: Physician Quality Reporting Initiative
PQRS: Physician Quality Reporting System
SMART: Specific, Measurable, Achievable, Relevant, Timely

## Footnotes

1
*Physician Quality Reporting System (PQRS) was initially founded as “Physician Quality Reporting Initiative” (PQRI).*

2
*Measurements for electronic health record reporting were not included*.
