# Supplementary material for: Characterization and effectiveness of pay-for-performance in ophthalmology: a systematic review
Source: BMC Health Serv Res. 2017 Jun 5;17:385. doi: 10.1186/s12913-017-2333-x (PMC5460462; doi:10.1186/s12913-017-2333-x)
Supplement: Additional file 1: — First results of search history (including duplicates), separated for single keyword combinations and for single databases. (DOCX 15 kb) [file 12913_2017_2333_MOESM1_ESM.docx]

**Table 3:** systematic comparison of *incentive elements* according to van Herck et al.

|  | **Incentive structure** | **Incentive size** | **Relation between Incentive structure and quality achievements** | **Frequency of incentive payment** | **Duration of incentive payments** | **Relative weights for quality indicators** | **Form of incentive structure** |
| --- | --- | --- | --- | --- | --- | --- | --- |
| **MedEncentive** | Bonus  Penalty | 10 % (bonus)  n.a. (penalty) | Absolute Reward | n.a. | Since 2004 | n.a. | Fixed amounts |
| **Kaiser Permanente Northern California** | Bonus | n.a. | Absolute Reward | n.a. | Since 1999 | No relative weights | Fixed amounts |
| **Physician Quality Reporting System (PQRS)** | Bonus  Penalty | +0.5 %-to +2.0 % (bonus)  -1.5 % (penalty) | Absolute Reward | n.a. | Since 2006 | Yearly adjustment of indicators and amount of payments | (Fixed amounts) |
| **ProvenCare** | Bundled payments | n.a. | Absolute Reward | n.a. | Since 2006 | n.a. | Fixed amounts |
